# The Push Forward in Rehabilitation: Validation of a Machine Learning Method for Detection of Wheelchair Propulsion Type

**DOI:** 10.3390/s24020657

**Published:** 2024-01-19

**Authors:** Rienk van der Slikke, Arie-Willem de Leeuw, Aleid de Rooij, Monique Berger

**Affiliations:** 1 Faculty of Health, Nutrition & Sport, The Hague University of Applied Sciences, 2521 EN The Hague, The Netherlands; a.w.deleeuw@hhs.nl (A.-W.d.L.); m.a.m.berger@hhs.nl (M.B.); 2 Department of BioMechanical Engineering, Delft University of Technology, 2628 CD Delft, The Netherlands; 3 Department of Innovation, Quality and Research, Basalt Revalidatie, 2545 AA The Hague, The Netherlands; a.derooij@basaltrevalidatie.nl; 4 Department of Orthopaedics, Rehabilitation and Physical Therapy, Leiden University Medical Center (LUMC), 2333 ZA Leiden, The Netherlands

**Keywords:** rehabilitation, inertial sensor, wheelchair activity

## Abstract

Within rehabilitation, there is a great need for a simple method to monitor wheelchair use, especially whether it is active or passive. For this purpose, an existing measurement technique was extended with a method for detecting self- or attendant-pushed wheelchair propulsion. The aim of this study was to validate this new detection method by comparison with manual annotation of wheelchair use. Twenty-four amputation and stroke patients completed a semi-structured course of active and passive wheelchair use. Based on a machine learning approach, a method was developed that detected the type of movement. The machine learning method was trained based on the data of a single-wheel sensor as well as a setup using an additional sensor on the frame. The method showed high accuracy (F1 = 0.886, frame and wheel sensor) even if only a single wheel sensor was used (F1 = 0.827). The developed and validated measurement method is ideally suited to easily determine wheelchair use and the corresponding activity level of patients in rehabilitation.

## 1. Introduction

The health benefits of a more active lifestyle are becoming increasingly clear, but so are the short- and long-term health dangers of insufficient exercise [1]. Yet, people with disabilities are significantly less (16–62%) likely to meet physical activity guidelines, making them more susceptible to inactivity-related health problems than the general population [2]. Lower-limb orthopedic patients in inpatient rehabilitation do much less physical activity than recommended in guidelines, especially in the elderly population [3]. In stroke patients, physical activity is recommended, yet most of the time patients spend their time sedentary [4], even if they can walk. For wheelchair-bound stroke or amputee patients, sedentary time will be even more prevalent. For the activity level of this target group, it is more important that they use the capacities they still have [5] and thus minimize the obviously worse health conditions that occur within this group [6]. So, given that they are wheelchair-bound, it is important that they push the wheelchair manually as often as possible, and that the conditions for this are optimized [7,8].

It is therefore essential to have a good understanding of the activity patterns of people with disabilities and to be able to give individuals insight into their physical activity behavior. However, there are only a few measurement methods that are easy to use and particularly focused on activity levels among manual wheelchair users (MWUs). A method that meets these requirements is a prerequisite for collecting more data on activity patterns within the MWU target group. This method should be applicable within both clinical and outpatient settings. With more insight into the physical activity behavior of wheelchair users in the different rehabilitation stages, more targeted advice can be given, allowing these patients to monitor their ability to follow the rehabilitation doctor’s advice at an individual level using the same measurement method.

Intervention studies regarding behavior change techniques (BCTs) [9] report tools like self-monitoring as effective elements [10] for changing activity patterns in people. Those BCTs rely on tools available for accurately monitoring physical activity, but for manual wheelchair users (MWUs), activity monitors are scarce, especially for quantifying free-living lifestyle behaviors. Although well-validated for wheelchair movement, the methods often show limited validity for distinguishing between self- and attendant-propulsion [11] if no body-worn sensors are incorporated. In a recent review by Lankhorst et al. [12], all sensor-based methods for measuring wheelchair use included body-worn sensors, with most of the studies aiming at spinal cord injury patients. Especially for certain MWU groups with alternative propulsion modes like one hand and one foot, detection validity might be compromised, typically leading to an overestimation of active time for MWUs [13]. In wheelchair sports, reliable inertial measurement unit (IMU)-based methods are available to measure mobility performance [14]. The accelerometers, gyroscopes, and magnetometers in these IMUs are used to measure wheelchair kinematics that are recalculated to wheelchair mobility performance characteristics, like distance traveled, speed, accelerations, turns, and so on. But with a focus on sports, this method has never been validated for distinguishing between self-pushing or being pushed. Once validated, this method could be very beneficial in rehabilitation, since it would provide important information about the activity level of wheelchair users by providing mobility characteristics over the course of the day, but then also whether it was performed by the user or an attendant.

The success of using a physical activity monitoring method depends on its ease of use and validity for the entire target group of MWUs. For ease of use, it is important that the instrumentation is as simple as possible, with as few sensors as possible, and preferably not worn on the body. For validity, it is important that the measurement system works well not only for the average wheelchair user but also for people with alternative, somewhat less conventional patterns in propulsion. Popp et al. [11] described multiple IMU-sensor configurations for detecting active or passive wheelchair use, with the best results if both the accelerometer and gyroscope data of multiple sensors were used (93% accuracy) compared to only accelerometer-based predictions (82%). Within an IMU, a selection can be made of which sensors are actively used, to match usage in terms of frequency and data to the application. Although still more power-consuming than accelerometers, due to recent technological advancement, the gyroscopes in a miniature IMU can easily be used for over a week. So, there is less limitation in using gyroscopes to measure wheelchair movement compared to previous studies like Popp [11]. If no body-worn sensors are used, the detection of active or passive propulsion will mainly focus on differences in wheelchair kinematics. Variation in movement (speed and rotational speed) is a good indication of active propulsion. But this is especially true for the more active wheelchair user. For the less active wheelchair user, low speeds and combinations of foot and hand propulsion lead to patterns that are less unambiguous and more difficult to interpret. Therefore, it is important to test wheelchair activity monitoring with actual wheelchair users with different and alternative propulsion patterns that represent the target group, since untrained wheelchair users show different propulsion patterns [15].

This research aims to validate a newly developed method for detecting self-or attendant-pushed wheelchair propulsion. The method should be able to detect active or passive propulsion in the toughest conditions, so for subjects with a variety of active propulsion techniques in free movement and tasks that are deemed challenging for detection algorithms. In a research setting, it is easy to use a more comprehensive measurement and analysis configuration, so for this investigation into the most appropriate method for measuring active or passive propulsion, multiple sensors were used. Moreover, the method for monitoring the physical activity of MWUs should be sensitive to all forms of propulsion. To be able to detect patterns that are less obvious and discern the most subtle differences, a range of machine learning methods was used. The best method regarding accuracy and ease of use, with minimal instrumentation and no body-worn sensors, could then be selected.

## 2. Materials and Methods

### 2.1. Design

This study design was cross-sectional and observational and could be categorized as instrumental, based on the classification as described by Montero et al. [16].

### 2.2. Subjects

Stroke and lower extremity amputation patients admitted to the Basalt Rehabilitation Centre were asked to participate in the study. Participants had to be mainly wheelchair-bound but sufficiently far along in the rehabilitation process to participate safely and responsibly. The inclusion criteria were over the age of 18 years, able to independently transfer safely from bed to wheelchair, and able to safely move around within the rehabilitation center.

A total of 24 in-patient MWUs (2 females, 22 males, age of 57.8 ± 12.7) participated in this study, of whom 11 people were recovering from a stroke (7 right-sided and 6 left-sided) and 13 after amputation (9 unilateral transtibial, 1 double transtibial and 1 unilateral transfemoral). Each participant signed a written informed consent after being informed about the procedure and goal of the research project, as described in the ethical approval, N21.178, by the Basalt Ethical Commission.

### 2.3. Device

Two Movesense HR+ sensors (Movesense, Vantaa, Finland) were placed in mounts fixed to the right wheel hub and the middle of the crossbars on the frame. The Movesense HR+ is a lightweight (10 g including coin cell battery), 10 DOF IMU that connects via Bluetooth to a mobile device running a dedicated app. The 3-axis gyroscope and the 3-axis acceleration data were retrieved at 50 Hz using the Movesense Showcase app (iOS version 1.1.0) from both synchronized sensors and stored as a JSON file on the mobile device for further analysis.

### 2.4. Measurement

**Figure 1:**
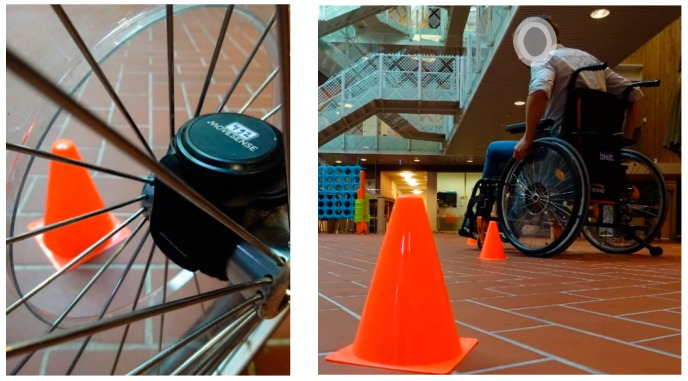
Mounted Movesense sensor and example of the test track.

Participants were picked up at their rooms, where the sensors were placed on the Quickie loaner wheelchair. They were escorted to an outdoor covered area where they had to perform a series of short wheelchair tests; see Figure 1. These tests consisted of segments of wheelchair motion that were deemed difficult for the distinction between self-pushing or being pushed, due to a lot of rotation and variation in speed. The parts consisted of wheelchair riding: 9 m slow, 9 m normal speed, 9 m slalom, and 9 m with 2 intermediate stops to almost standstill. These tests were performed both by the attendant pushing the wheelchair and by the participant pushing himself. The participants were asked which propulsion modes they used in daily life and those modes were included in the different tests. So, some stroke patients used 1 hand and 1 foot, whereas one amputee preferred to drive backward with foot (prosthetic leg) propulsion. Based on the propulsion techniques used, the number of tests performed slightly differed. After the more standardized tests, the participants drove around the rehab center in different propulsion modalities and on different surfaces, finishing in their own rooms again. The measurements took ~25 min. The supervisor manually kept track of times for the different propulsion modalities, during the tests and in the free movement through the rehabilitation center.

### 2.5. Data Processing

Raw sensor data were processed in a custom-built Python (Python 3.10.13) script to retrieve wheelchair motion as described by van der Slikke [14] and the modified configuration with fewer sensors described by Rupf [17] and van Dijk [18]. This Python script converted the JSON files to IMU data time series that were used to calculate basic wheelchair kinematics like wheelchair speed, distance traveled, and frame rotation, taking the wheelchair dimensions, such as wheel diameter and track width, into account. To develop the algorithm that can distinguish between active and passive wheelchair use, wheelchair movement was divided into contiguous segments of conditional velocity above 0.1 m/s, with segments below 0.1 m/s or of a duration shorter than 2 s disregarded [11] for further analysis. Each segment was labeled active (self-propelled), or passive (pushed by an attendant), based on the manual timed annotations by the test supervisor.

**Figure 2:**
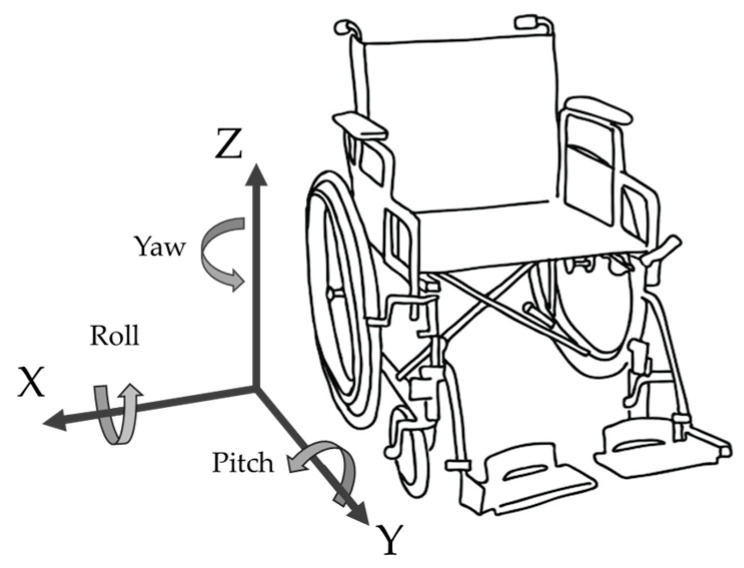
Wheelchair and sensor reference frame. This sensor reference frame applies when the sensor is on top of the axis, in the right wheel.

Wheelchair-mounted sensors provide a substantial range of features available to help in distinguishing the different forms of propulsion. The key feature would be forward speed and derivatives (distance, variation in speed, acceleration), but also, changes in direction could be relevant, especially in single-hand propulsion. Therefore, frame rotation is also included as a potential feature. To obtain both IMU-based forward and frame rotational speed, multiple sensor configurations are possible. The most accurate is a configuration with *one* wheel-mounted IMU and *one* frame-mounted IMU, but also a single wheel-mounted IMU suffices, albeit less accurately [18]. For this research, the results of the single wheel-mounted sensor are described in the “wheel model” (S1; see Figure 2), whereas the results of the model that also incorporates the data of the additional frame-mounted sensor is referred to as the “full model” (S2). The results of both configurations and models will be compared to provide insight into the possible additional value of the second sensor mounted on the frame. Minor offset shifts of the raw gyroscope were corrected by subtracting the mean gyroscope signal values for periods of no motion (<5°/s). Raw gyroscope and accelerometer features of wheel and frame sensors were used as predictors for sensor-based classification of segments. Forward speed is derived from the wheel gyroscope, whereas frame rotational speed is derived from the frame sensor (S2) or solely based on the wheel sensor using the AHRS algorithm (S1) [17], splitting the sensor movement to wheel roll, pitch, and yaw; see Figure 2.

An aggregation-based approach [19,20] was employed to turn these quantities into predictor variables. Hence, the predictors are aggregates and summarize the values of one of the quantities at all time points in a segment. For example, a predictor is the median of all values of the angular velocity of the wheel sensor around the yaw axis in a segment. In the approach used, it is important to only have a moderate set of predictors, as having too many predictors might negatively influence the generalizability of the model. Therefore, only the median, standard deviation, skewness, and kurtosis are used as aggregate functions [19].

### 2.6. Performance Analysis

The performance of the models was described in accuracy, precision, recall and F1 score [21], based on the true positive (TP), true negative (TN), false positive (FP), and false negative (FN). The following definitions were adopted:

$$Accuracy=\frac{TP+TN}{TP+TN+FP+FN}$$

$$Precision=\frac{TP}{TP+FN}$$

$$Recall=\frac{TP}{TP+FN}$$

$$F1=2\times \frac{Precision\times Recall}{Precision+Recall}$$

### 2.7. Machine Learning

Several machine learning techniques were used to construct models connecting the predictor variables to the binary wheelchair use, with 0 for passive and 1 for active. A priori, it is unknown which modeling techniques will perform best, and therefore the accuracy of the most common binary classification algorithms was examined: Logistic Regression [22], Support Vector Machine [23], Random Forest [24], Naive Bayes [25], and XGBoost [26].

**Figure 3:**
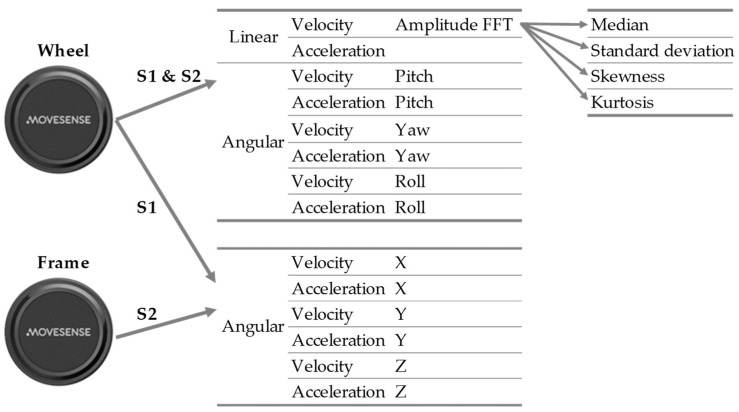
Overview of the predictor variables used in both models. In total, 8 input variables times 4 aggregate functions are derived from the wheel-mounted sensor for both models (S1 and S2), and an additional 6 × 4 variables are derived from raw gyroscope sensor data from the wheel-mounted sensor (S1) or frame-mounted sensor (S2).

Based on the sensor configurations (S1 and S2; see Figure 3), two models were constructed. In both models, features describing the linear speed and acceleration of the wheelchair, and the angular velocities and accelerations around the roll, pitch, and yaw axes (Figure 3), were considered. Depending on whether the full or wheel model is considered, values of the angular velocities and accelerations directly obtained from the gyroscope of the wheel sensor (S1), or frame sensor (S2) were used. A Fast Fourier Transformation (FFT) was applied to decompose the linear speed into a Fourier series, of which the amplitudes of the coefficients were used as inputs for the model. Additionally, the three-dimensional values of the angular velocities and accelerations directly obtained from the gyroscope of the wheel sensor (S1) or frame sensor (S2) were used. In total, 56 predictor variables were used in both models, but their signal origin differed.

Before the model training, all highly correlated features (|r| > 0.7) were removed to avoid multicollinearity [27], resulting in 38 remaining predictor values for the S1 and 42 for the S2 model. The data set was randomly split into training and test sets containing 80% and 20% of all the data, respectively. To ensure equal distribution over the target variable and cause of disability (stroke or amputation), stratified sampling was used to ensure that the models were equally valid for both types of patients.

The training set was used for model selection and parameter tuning by applying 10-fold cross-validation and using the F1 score as the optimization criterion [21]. Then, the entire training data set was used to construct the final model for predictions for each data point in the test set. These predictions were compared to the actual values, and the performance of the models was then assessed by determining the accuracy, precision, recall, and F1 score [21]. Finally, the model was constructed on all the data to determine the feature importance of the predictor variables [28]. In short, the feature importance score of a predictor variable is a value between 0 and 1, indicating its contribution to improving the performance of the model, with 1 being the most important. Like the data processing, the modeling was done in a custom-written Python script, using the packages *sklearn* and *xgboost.*

## 3. Results

### 3.1. Kinematic Data

**Figure 4:**
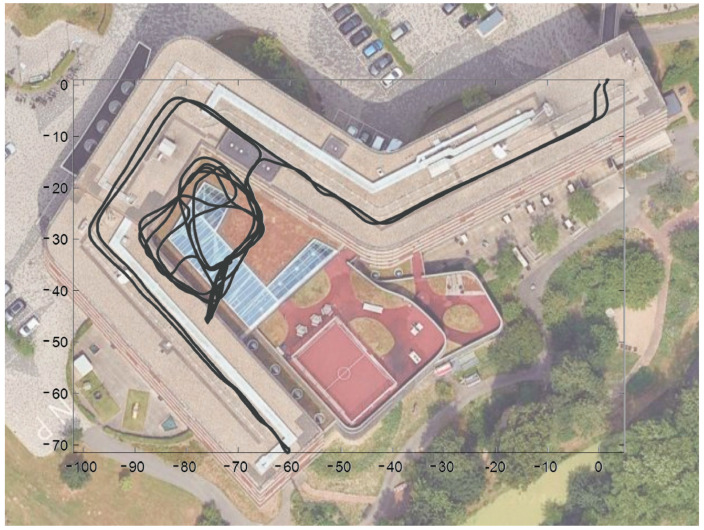
Path reconstructed from IMU data, displayed on an overlay of the rehabilitation center (Google Maps, 2023). The movement from the room to the elevator is shown, followed by the test and free movement in the covered outdoor area, and finally free movement on the upper floor corridor and return to the room.

Twenty-four measurements were taken, with an average duration of 23.8 min (SD = 4.5 min), during which the participants traveled 782.5 m (±203.8 m), with an average speed of 0.86 m/s (±0.10 m/s). A typical example of the measurement as reconstructed from the sensor-based kinematic wheelchair data is shown in Figure 4, with the track from the participant’s room to the covered area where the protocolled test was performed.

**Figure 5:**
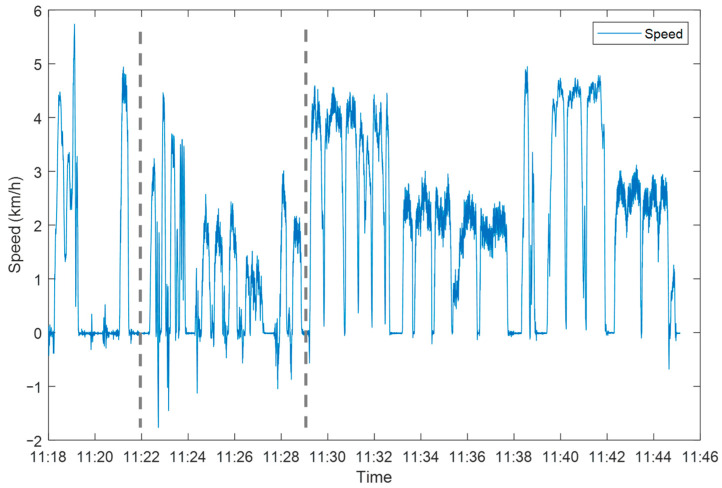
Speed plot of a typical measurement, with the first section (<11:22) representing the movement to the test site, the two parts of the test (active–passive), and the free motion (11:29>) within the rehabilitation center and return to the room.

A typical speed profile of the measurements is shown in Figure 5, with a clear distinction between the different aspects of the protocol, both the short test sections as well as the transfer to the test site and free movement.

### 3.2. Segments

The data of all 24 participants contained 949 segments, of which 527 were classified as active and 420 as passive, with an almost equal division between the segments of stroke and amputation patients. For the active segments, there are slightly fewer examples from stroke patients (250 vs. 277), and, for the passive segments, there are 210 examples for both stroke and amputation patients.

**Figure 6:**
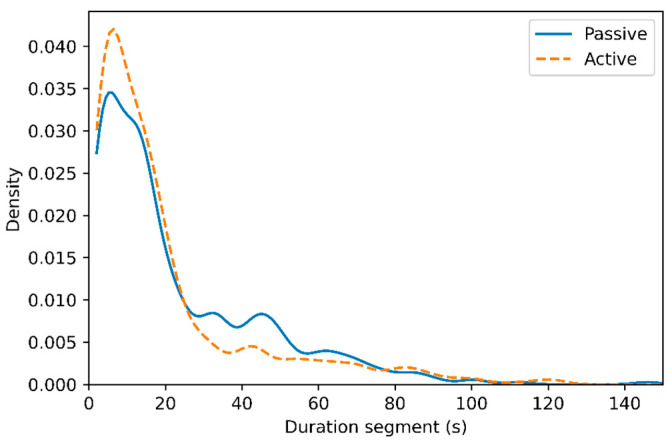
Density distribution of the segment duration of passive (solid) and active (dashed) wheelchair use, showing that the protocol used produces mostly short segments.

The distribution of duration for active and passive segments is shown in Figure 6, with a very similar distribution for both types. The testing protocol focused on shorter activities, and this is also visible in the duration of the segments, with mainly segment durations of less than 20 s.

### 3.3. Classification of Wheelchair Use with Machine Learning

For both models (wheel (S1) and full model (S2)), 25 different random splits of the training and test sets were considered to account for potential randomization errors. The model and corresponding parameters that performed best across the different random splits were selected. In both models, the best-performing model was an XGBoost classifier. The model parameters were set to the default values as specified in the XGBoost 1.7.5 package. The model performance is summarized in Table 1, with the F1 scores all above 0.8, but with the full model slightly outperforming the wheel model.

### 3.4. Feature Importance

The importance of the different predictors in the wheel model is displayed in Table 2, showing median angular acceleration and velocity around the roll axis as the most dominant. Since the correlation with the target variable is negative, larger values are associated with passive wheelchair use. The standard deviation of the linear acceleration and amplitudes of the Fourier series for the linear speed are important as well. There are numerous predictor variables with non-negligible importance scores, indicating that most of the collected information is relevant for classifying active and passive wheelchair use.

## 4. Discussion

To aid in rehabilitation application, an easy-to-use measurement method with valuable wheelchair mobility performance outcomes was extended with the detection of active or passive wheelchair use, validated in this study. To enhance its use in a wide range of rehabilitation patients, tough conditions were applied in the validation, requiring a more sophisticated approach like machine learning. Using this method, results show a valid detection of active or passive wheelchair use, even based on single-wheel sensor data. This method is therefore applicable in daily practice, can be used to build reference databases for activity levels in MWUs, and is applicable as an intervention tool for individual monitoring of activity. Patients frequently appear to underestimate or overestimate their physical activity levels [29]. Monitoring and feedback are recognized as potential effective behavior change techniques (BCTs) for promoting and augmenting physical activity [9], which can be supported by the use of the method presented in this study.

The average speed of the subjects in self-propulsion was lower than previously reported in home situations by Tolerico [30], who reported an average speed of 0.79 (±0.19). In this study, the subjects with amputation had a median speed of 0.68 m/s (±0.31) and the stroke subjects 0.56 m/s (±0.29), indicating the less intense propulsion styles in these groups. This lower average speed and the observed variety in propulsion modes indicate that the participants were indeed selected with propulsion modes that were challenging for automatic detection. Although the measurement duration was shorter than the study of Popp et al. [11], nearly all the measurement time consisted of forward movement, effectively resulting in more movement time for building the model and validation, contributing to better modeling. Although a reasonable number of patients were included in this study, with similar measurement times for modeling as shown in previous studies, there are still some limitations that need mentioning. All the measurements were performed with the wheelchair provided by the rehabilitation center, so all were of a similar type. Furthermore, only a small variation in surfaces was included, so no grass, cobblestone, or other pavement. These limitations are expected to affect study outcomes only marginally.

Previous studies reported overall agreement of 82.1% [31], 89.3% [32], and up to 81–95% [33] between detected propulsion modes, but always with also a hand- or wrist-worn sensor. In this study, the detection methods show similar validity for the two-sensor configuration (full model), but already good validity with only a single-wheel sensor. For ease of use, the single-wheel sensor model is best and provides sufficiently valid detection. The major advantage of a single sensor over a multiple-sensor configuration is the lack of the need for synchronization. This is not only technically easier, but it often leads to cheaper solutions and the ability to log data on the sensor without connecting to a mobile device or another sensor. The Movesense sensors used in this study are now also available with onboard logging functionality for multiple days. If even more accuracy is required, the full model with an additional frame sensor does provide slightly better results (F1 score of 0.827 vs. 0.886). These results are similar to previous studies [11], but the sensor configuration (especially S1) is simpler and only on the wheelchair, so the practical applications are better.

The use of different machine learning models made it possible to investigate which model produced the best results. The machine learning models gave the advantage that many aspects of wheelchair movement could be included in the model without bias. The possible interactions between the predictors and any non-linear relationships were also modeled. The number of predictor variables included is substantive, with expected overlap between variables. Forward speed and acceleration, for example, are likely to have a close relationship with angular velocity and acceleration around the roll axis. Therefore, all highly correlated features (|r| > 0.7) were removed to avoid multicollinearity [27], which resulted in dropping quite a few variables for further analysis (20 for S1 and 14 for S2). But otherwise, no selection was made in advance to let the model itself determine the most relevant variables. The importance is quite similar for the top five most important predictor variables (see Table 2), indicating that there is no single variable well-discriminating between passive and active propulsion. Hence, a non-machine learning approach based on simpler algorithms is less likely to show similar results. The final machine learning model is included in the Supplementary Materials for future use.

The developed method will be used for current and future research projects within the Basalt Rehabilitation Centre, but, with the model being available, it could also be employed by other researchers. From a practical perspective, the method still requires some technical support, with data collection in the Movesense Showcase app and post-measurement analysis in a Python script, but it is likely that in the foreseeable future, these measurements can be conducted in a more user-friendly way. Already this research group is working on easy-to-use data logging of wheelchair mobility performance, in which this model for active–passive propulsion is incorporated. The option to log the sensor onboard for multiple days will increase the scope of applications.

## 5. Conclusions

The developed machine learning-based model for detecting passive or active wheelchair use proves valid, even with the simplest instrumentation, namely a single wheel-mounted inertial sensor. With this addition, the wheelchair mobility performance monitor now offers a comprehensive tool for monitoring wheelchair kinematics, not only in sports but also applicable in clinical practice in rehabilitation. This enables more research into the activity patterns in wheelchair use and can eventually serve as a tool for individual monitoring and behavioral change towards a more active lifestyle within safe boundaries.

## Figures and Tables

**Table 1:** Average performance of the wheel and full models on the test set across 25 different random splits of the total data. The performance of the full model is slightly better than the performance of the wheel model.

	Accuracy	Precision	Recall	F1 Score
Wheel model (S1)	0.805	0.836	0.819	0.827
Full model (S2)	0.873	0.888	0.886	0.886

**Table 2:** Overview of the five most important predictor variables of the wheel model. The importance of a predictor variable is assessed by determining its contribution to improving the performance of the model. The sum of all importance scores is equal to 1, and larger values indicate more important predictor variables. The correlation with the target variable is shown, defined as 0 and 1 for passive and active wheelchair use, respectively.

Predictor Variable	Importance Score	Correlation Target
Median angular acceleration around the roll axis	0.06	−
Median angular velocity around the roll axis	0.06	−
Standard deviation linear acceleration	0.05	+
Standard dev. amplitudes of Fourier series for linear speed	0.05	−
Kurtosis angular velocity y-component of wheel sensor	0.05	+

## Data Availability

Due to privacy reasons, data are not available as separate measurements, but the core outcomes are available as a base for the model provided in the Supplementary Materials.

## Supplementary Materials

The following supporting information can be downloaded at: https://www.mdpi.com/article/10.3390/s24020657/s1, Python script and model files: File S1: WheelModel.csv.; File S2: FullModel.csv.; File S3: Scriptmodel.py.

## References

[1] Rhodes R.E., Janssen I., Bredin S.S.D., Warburton D.E.R., Bauman A. (2017). Physical activity: Health impact, prevalence, correlates and interventions. Psychol. Health.

[2] Ginis K.A.M., van der Ploeg H.P., Foster C., Lai B., McBride C.B., Ng K., Pratt M., Shirazipour C.H., Smith B., Vásquez P.M. (2021). Participation of people living with disabilities in physical activity: A global perspective. Lancet.

[3] Peiris C.L., Taylor N.F., Shields N. (2013). Patients receiving inpatient rehabilitation for lower limb orthopaedic conditions do much less physical activity than recommended in guidelines for healthy older adults: An observational study. J. Physiother..

[4] de Jong A.U., Smith M., Callisaya M.L., Schmidt M., Simpson D.B. (2021). Sedentary time and physical activity patterns of stroke survivors during the inpatient rehabilitation week. Int. J. Rehabil. Res..

[5] Ellapen T.J., Hammill H.V., Swanepoel M., Strydom G.L. (2017). The health benefits and constraints of exercise therapy for wheelchair users: A clinical commentary. Afr. J. Disabil..

[6] Cowan R.E., Silveira S.L., Helle T., Læssøe U., Gøeg K.R., Bangshaab J., Motl R.W. (2022). Lifestyle physical activity in manual wheelchair users—An overlooked public health opportunity. Spinal Cord.

[7] de Groot S., Post M.W.M., Bongers-Janssen H.M.H., Bloemen-Vrencken J.H., van der Woude L.H.V. (2011). Is manual wheelchair satisfaction related to active lifestyle and participation in people with a spinal cord injury?. Spinal Cord.

[8] Warms C.A., Whitney J.D., Belza B. (2008). Measurement and description of physical activity in adult manual wheelchair users. Disabil. Health J..

[9] Michie S., Ashford S., Sniehotta F.F., Dombrowski S.U., Bishop A., French D.P. (2011). A refined taxonomy of behaviour change techniques to help people change their physical activity and healthy eating behaviours: The CALO-RE taxonomy. Psychol. Health.

[10] Jaarsma E.A., Smith B. (2018). Promoting physical activity for disabled people who are ready to become physically active: A systematic review. Psychol. Sport Exerc..

[11] Popp W.L., Brogioli M., Leuenberger K., Albisser U., Frotzler A., Curt A., Gassert R., Starkey M.L. (2016). A novel algorithm for detecting active propulsion in wheelchair users following spinal cord injury. Med. Eng. Phys..

[12] Lankhorst K., Oerbekke M., Berg-Emons R.v.D., Takken T., de Groot J. (2020). Instruments Measuring Physical Activity in Individuals Who Use a Wheelchair: A Systematic Review of Measurement Properties. Arch. Phys. Med. Rehabil..

[13] Nightingale T.E., Rouse P.C., Thompson D., Bilzon J.L.J. (2017). Measurement of Physical Activity and Energy Expenditure in Wheelchair Users: Methods, Considerations and Future Directions. Sports Med. Open.

[14] van der Slikke R., Berger M., Bregman D., Lagerberg A., Veeger H. (2015). Opportunities for measuring wheelchair kinematics in match settings; reliability of a three inertial sensor configuration. J. Biomech..

[15] Symonds A., Holloway C., Suzuki T., Smitham P., Gall A., Taylor S.J. (2016). Identifying key experience-related differences in over-ground manual wheelchair propulsion biomechanics. J. Rehabil. Assist. Technol. Eng..

[16] Montero I., León O.G. (2007). A Guide for Naming Research Studies in Psychology. Int. J. Clin. Health Psychol..

[17] Rupf R., Tsai M., Thomas S., Klimstra M. (2021). Original article: Validity of measuring wheelchair kinematics using one inertial measurement unit during commonly used testing protocols in elite wheelchair court sports. J. Biomech..

[18] Van Dijk M.P., van der Slikke R.M., Rupf R., Hoozemans M.J., Berger M.A., Veeger D.H. (2022). Obtaining wheelchair kinematics with one sensor only? The trade-off between number of inertial sensors and accuracy for measuring wheelchair mobility performance in sports. J. Biomech..

[19] De Leeuw A.-W., van der Zwaard S., van Baar R., Knobbe A. (2022). Personalized machine learning approach to injury monitoring in elite volleyball players. Eur. J. Sport Sci..

[20] De Leeuw A.-W., van Baar R., Knobbe A., van der Zwaard S. (2022). Modeling Match Performance in Elite Volleyball Players: Importance of Jump Load and Strength Training Characteristics. Sensors.

[21] Sokolova M., Lapalme G. (2009). A systematic analysis of performance measures for classification tasks. Inf. Process. Manag..

[22] Cox D.R. (1958). The Regression Analysis of Binary Sequences. J. R. Stat. Soc. Ser. B Methodol..

[23] Cortes C., Vapnik V. (1995). Support-vector networks. Mach. Learn..

[24] Breiman L. (2001). Random forests. Mach. Learn..

[25] Webb G.I., Keogh E., Miikkulainen R. (2010). Naïve Bayes. Enc. Mach. Learn..

[26] Chen T., Guestrin C. XGBoost: A Scalable Tree Boosting System. Proceedings of the KDD ’16: 22nd ACM SIGKDD International Conference on Knowledge Discovery and Data Mining.

[27] Dormann C.F., Elith J., Bacher S., Buchmann C., Carl G., Carré G., Marquéz J.R.G., Gruber B., Lafourcade B., Leitão P.J. (2013). Collinearity: A review of methods to deal with it and a simulation study evaluating their performance. Ecography.

[28] Hastie T., Tibshirani R., Friedman J.H., Friedman J.H. (2009). The Elements of Statistical Learning: Data Mining, Inference, and Prediction.

[29] Adams S.A., Matthews C.E., Ebbeling C.B., Moore C.G., Cunningham J.E., Fulton J., Hebert J.R. (2005). The Effect of Social Desirability and Social Approval on Self-Reports of Physical Activity. Am. J. Epidemiol..

[30] Tolerico M.L., Ding D., Cooper R.A., Spaeth D.M., Fitzgerald S.G., Cooper R., Kelleher A., Boninger M.L. (2007). Assessing mobility characteristics and activity levels of manual wheelchair users. J. Rehabil. Res. Dev..

[31] Leving M.T., Horemans H.L.D., Vegter R.J.K., de Groot S., Bussmann J.B.J., van der Woude L.H.V. (2018). Validity of consumer-grade activity monitor to identify manual wheelchair propulsion in standardized activities of daily living. PLoS ONE.

[32] Hiremath S.V., Intille S.S., Kelleher A., Cooper R.A., Ding D. (2015). Detection of physical activities using a physical activity monitor system for wheelchair users. Med. Eng. Phys..

[33] Postma K., van Den H.J.G.B.-E., Bussmann J.B.J., Sluis T.A.R., Bergen M.P., Stam H.J. (2005). Validity of the detection of wheelchair propulsion as measured with an Activity Monitor in patients with spinal cord injury. Spinal Cord.

